# Effect of healthy diet and exercise on chemotherapy completion rate in women with breast cancer: The Lifestyle, Exercise and Nutrition Early after Diagnosis (LEANer) study: Study protocol for a randomized clinical trial

**DOI:** 10.1016/j.cct.2021.106508

**Published:** 2021-07-16

**Authors:** Tara Sanft, Maura Harrigan, Brenda Cartmel, Leah M. Ferrucci, Fang-Yong Li, Courtney McGowan, Michelle Zupa, Thai Hien Nguyen, Jennifer Ligibel, Marian L. Neuhouser, Dawn L. Hershman, Karen Basen-Engquist, Beth Jones, Tish Knobf, Anees Chagpar, Andrea Silber, Melinda L. Irwin

**Affiliations:** a Yale University School of Medicine, New Haven, CT, United States of America; b Yale University School of Public Health, New Haven, CT, United States of America; c Yale Cancer Center, New Haven, CT, United States of America; d Dana-Farber Cancer Institute, Boston, MA, United States of America; e Fred Hutchinson Cancer Research Center, Seattle, WA, United States of America; f Columbia University Medical Center, New York, NY, United States of America; g The University of Texas MD Anderson Cancer Center, Houston, TX, United States of America; h Yale School of Nursing, New Haven, CT, United States of America

**Keywords:** Breast cancer, Chemotherapy completion, Nutrition, Exercise, Lifestyle, Relative dose intensity

## Abstract

**Background:**

The World Cancer Research Fund and the American Cancer Society provide nutrition and physical activity guidelines for cancer survivors. Many women with breast cancer do not follow these guidelines and delay efforts toward following them until active treatment is complete. However, adoption of these recommended lifestyle behaviors soon after diagnosis may prevent adverse treatment-related side effects and may improve adherence to treatment, resulting in improved breast cancer prognosis. The Lifestyle, Exercise, and Nutrition Early after Diagnosis (LEANer) study is testing the effect of a nutrition and physical activity intervention on chemotherapy completion rates.

**Methods:**

172 women with stage I-III breast cancer undergoing chemotherapy will be randomized 1:1 to a yearlong, 16 session, nutrition and exercise intervention or usual care control group. The intervention is delivered by registered dietitians specializing in oncology nutrition and exercise training. The intervention includes goal setting to meet nutrition and physical activity guidelines for cancer survivors. After each chemotherapy session, date and dose of each drug administered, and reason for dose-adjustments and/or dose-delays are abstracted from the electronic medical record or obtained from the treating oncologist. Chemotherapy completion rate is assessed as the average relative dose-intensity (RDI) for the originally planned regimen based on standard formulas. Secondary endpoints of endocrine therapy adherence, treatment-related side effects, and changes in inflammatory and metabolic biomarkers, body composition, and patient reported outcomes are assessed at four timepoints.

**Discussion:**

If successful, this study has the potential to make healthy lifestyle interventions a standard component of breast cancer treatment.

## 1. Introduction

For lowering risk of breast cancer recurrence and mortality, the World Cancer Research Foundation (WCRF), the American Institute for Cancer Research (AICR), and the American Cancer Society (ACS) recommend following a dietary pattern that is high in vegetables, fruits, and whole grains; and an exercise regimen which includes 150 min per week of moderate-intensity exercise or 75 min of vigorous-intensity exercise plus two strength training sessions per week [1-3].

A growing number of studies have evaluated the impact of following the recommended lifestyle behaviors on cancer risk and mortality [4-7]. For instance, the Vitamins and Lifestyle (VITAL) study showed that breast cancer risk was reduced by 60% in women who met the WCRF/AICR recommendations compared with those who did not meet the recommendations [5]. To our knowledge, no trial has examined in women newly diagnosed with breast cancer the effect of practicing the dietary and physical activity guidelines on chemotherapy completion rate and endocrine therapy adherence [8].

Roughly 12–28% of breast cancer patients who initiate adjuvant chemotherapy discontinue early [9,10]. Receipt of <85% of the prescribed dose of chemotherapy, which may be due to either early discontinuation or dose reduction, is associated with worse relapse-free and overall survival [11-14].

Few studies have examined how lifestyle behaviors may impact chemotherapy completion rates as a primary aim. We recently completed a retrospective chart review of body mass index (BMI) and physical activity on chemotherapy completion rate in 243 women receiving chemotherapy. Physical activity was associated with completion of chemotherapy (OR 7.6, 95% CI 1.4–41.2, *P* = 0.02) [15].

There is a need to more fully evaluate the role of adherence to nutrition and physical activity guidelines on chemotherapy completion rates. The Lifestyle, Exercise And Nutrition Early after Diagnosis (LEANer) study is designed to test the effect of a nutrition and physical activity intervention on rates of chemotherapy completion and adherence to endocrine therapy, as well as treatment-related side effects and changes in biomarkers, body composition and patient-reported outcomes, in women with early stage breast cancer.

## 2. Materials and methods

### 2.1. Primary aim

**Fig. 1.**
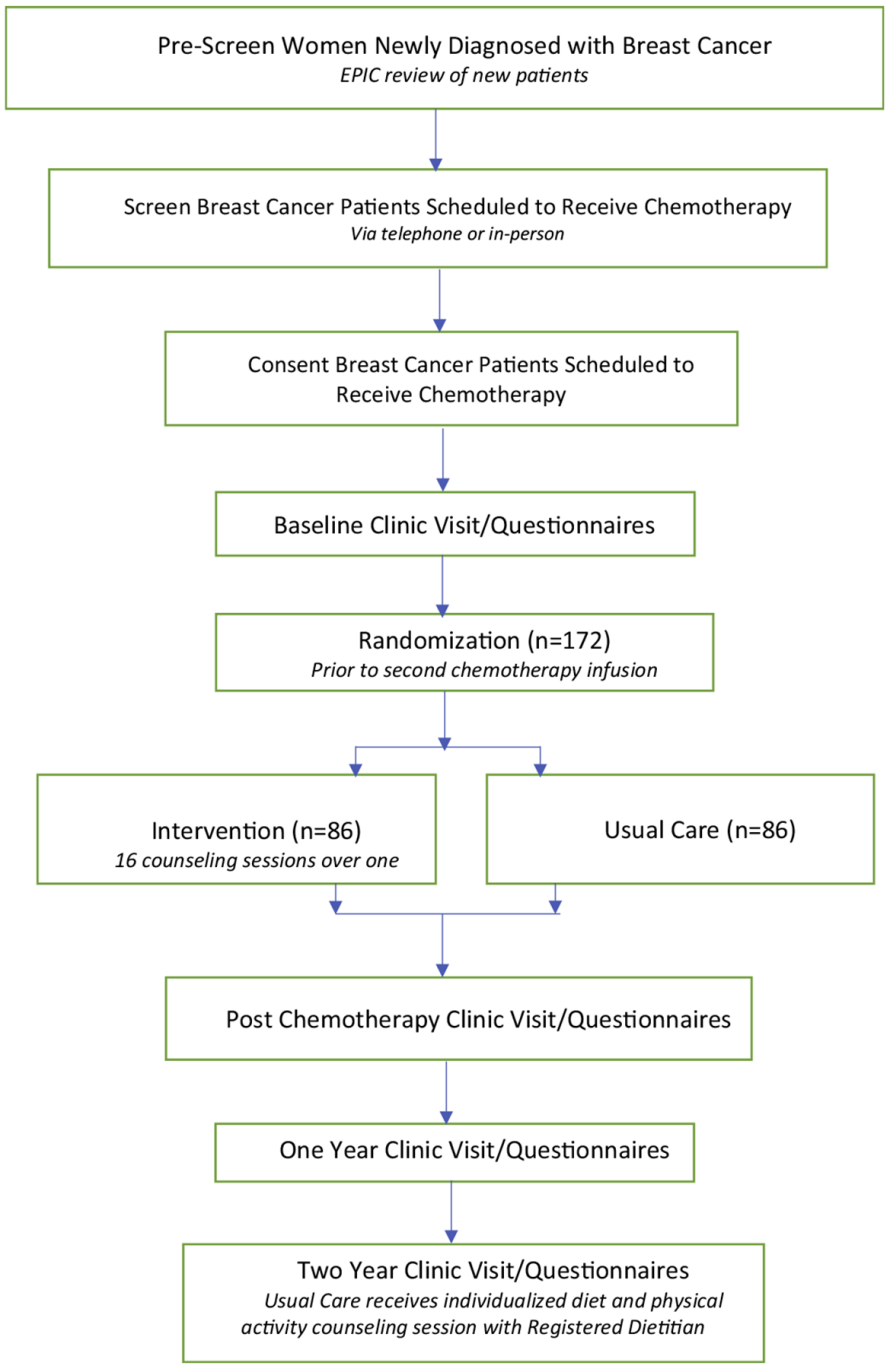
The Lifestyle, Exercise, and Nutrition Early after Diagnosis (LEANer) study schema.

LEANer is a randomized controlled trial investigating the effect of a nutrition and physical activity intervention vs. usual care on chemotherapy completion rates in women with early stage breast cancer (Fig. 1).

### 2.2. Secondary aims

Secondary aims include examining the impact of the intervention vs. usual care on endocrine therapy adherence, body composition, serum biomarkers, treatment-related side effects and patient-reported outcomes.

### 2.3. Design

**Fig. 2.**
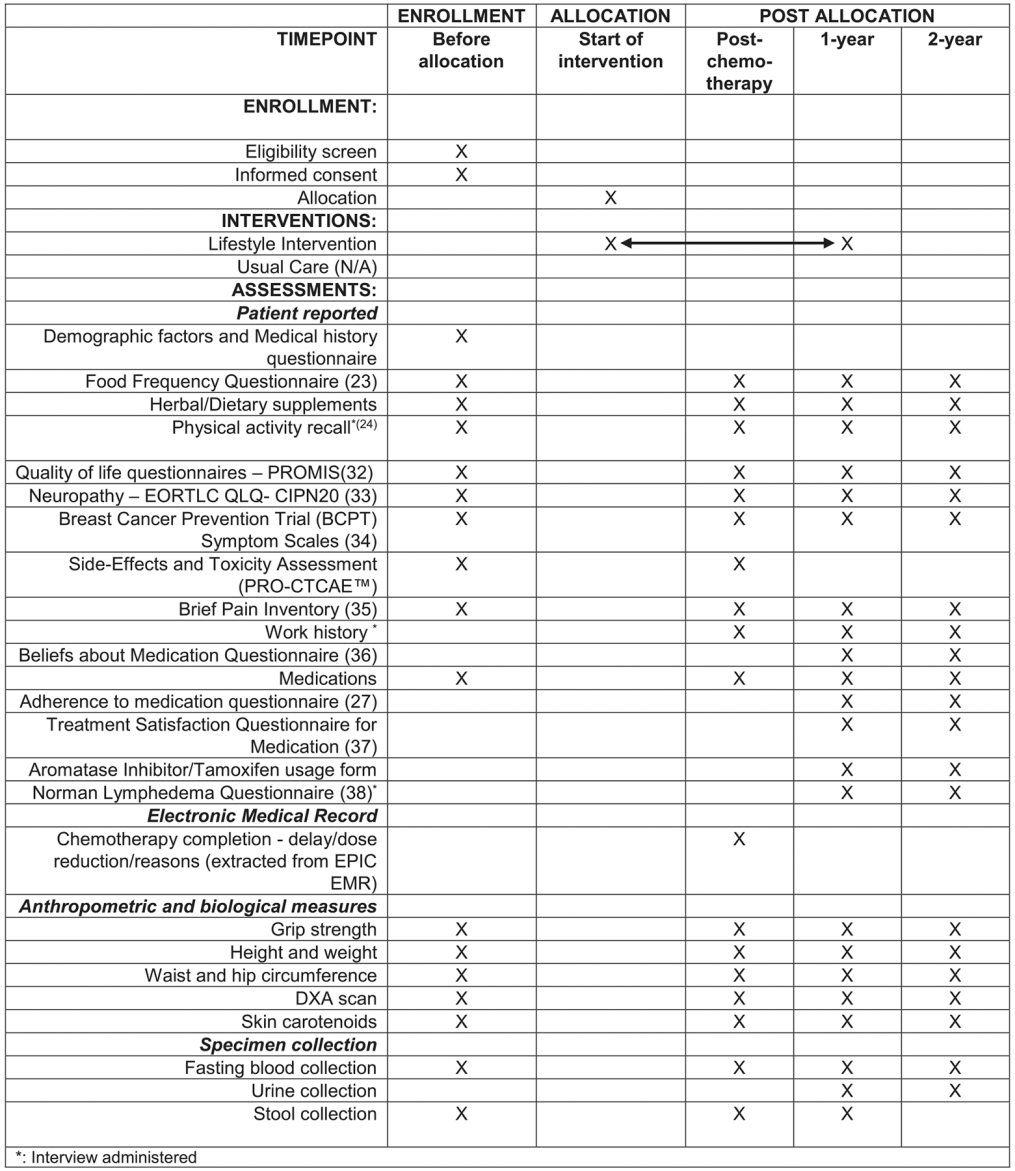
The LEANer study schedule of enrollment, interventions, and assessments.

Target enrollment is 172 women with stage I-III breast cancer undergoing chemotherapy (neoadjuvant or adjuvant) for curative intent. The trial funder is the National Cancer Institute (NCI). Participants are randomized 1:1 to a 12 month in-person and telephone-based intervention or a usual care control group. Usual care was selected as a comparator as it limits contact with study personnel and thus minimizes change in behavior due to study participation. Patients are enrolled through the Smilow Cancer Hospital Network at Yale-New Haven Hospital and through the Dana-Farber Cancer Institute in Boston, MA. The intervention is delivered in-person, by telephone or via a combination of in-person and telephone-based sessions by the Yale-based registered dietitians (RDs). The trial aims for robust minority enrollment with study team members playing leadership roles focused on diversity and clinical trial enrollment, and partnerships with clinicians whose practices predominantly serve minority populations. The study enrollment, interventions and assessments are shown in Fig. 2. The Yale Human Investigation Committee (IRB) and the Dana-Farber Cancer Institute Institutional Review Board approved informed consent and all relevant procedures. Informed consent will be obtained from all participants.

### 2.4. Eligibility

Women with stage I-III breast cancer undergoing neoadjuvant or adjuvant chemotherapy who are able to walk and not meeting the recommended diet and physical activity guidelines are eligible (Table 1).

### 2.5. Screening questionnaire

Eligibility criteria are determined from electronic medical records (EMR) and via interview-administered questionnaires, which are conducted following the in-person or telephone consent discussion, conducted by one of the study staff, and receipt of informed consent. Screening includes determining eligibility on diet and physical activity by asking the following questions: 1. ‘Prior to your diagnosis of breast cancer, did you exercise, (for example, jogging, brisk walking for exercise or stationary bicycling)?’ If the patient reports exercising less than 150 min per week of moderate-intensity aerobic exercise or less than 75 min per week of vigorous-intensity aerobic exercise or an equivalent combination they are coded ‘no’ on the exercise criteria; 2a. ‘Prior to your diagnosis of breast cancer, how many servings of vegetables did you usually eat per day? and 2b. ‘Prior to your diagnosis of breast cancer, how many servings of fruit did you usually eat per day?’ Eligibility criterium for fruit and vegetable intake: less than 7 fruit and vegetable servings combined per day. If the patient reports eating less than 7 fruits and vegetable servings per day, they are coded ‘no’ on the healthy eating criteria. To be eligible, participants need to respond ‘No’ to both questions. This simple question approach has been endorsed by others [16] and we have used it as screening criteria in our other nutrition and physical activity trials [17].

### 2.6. Randomization and stratification

Eligible women are randomized to either intervention or usual care in a 1:1 ratio, stratified by HER2 status (+ or −), Hormone Receptor (HR + or −) status, and number of chemotherapy cycles for HER2 negative patients (4 cycles or 5 or more). In total six strata are used:

- HER2−, HR +, 4 cycles
- HER2−, HR +, 5 or more cycles
- HER2−, HR−, 4 cycles
- HER2−, HR−, 5 or more cycles
- HER2 +, HR +
- HER2 +, HR−

The randomization list was generated for each stratum by computer using the permutation method with variable block sizes. After completion of the baseline assessments, eligible patients are randomized by a member of the Yale research staff. Sealed envelopes containing the random allocation are prepared by an individual who is not affiliated with the study and opened at the point of randomization. The individuals conducting the dual energy X-ray absorptiometry (DXA) scan and the laboratory biomarker analysis are blinded to the randomization group.

## 3. Study intervention

### 3.1. Setting

Women can be enrolled and initiate the intervention any time prior to their second chemotherapy. The intervention schedule of sixteen 30-min sessions over 12 months follows the participant through the trajectory of their active treatment (3 times in the first month, 2 times each month for months 2 through 5, and 5 times over the next 6 months). Our previous Lifestyle, Exercise and Nutrition (LEAN) trial, conducted in the post-treatment setting, compared in-person counseling to telephone-counseling and found no between-group difference in intervention adherence, with both the in-person and telephone-based intervention groups experiencing similar favorable changes in physical activity, diet, body weight and serum biomarkers [18]. Thus, for the LEANer study, all intervention sessions can be completed either in-person or by telephone, as per participant preference or as determined by study site location and COVID-19 restrictions. Independent of study arm, all concomitant care and interventions are permitted during the study intervention (year 1) and follow-up (year 2).

### 3.2. Content of counseling sessions

The intervention is based on the LEAN protocol [18], which was developed by Drs. Irwin, Sanft and Ms. Harrigan, adapted from the Diabetes Prevention Program [19] and further adapted to meet the unique concerns of breast cancer patients in active treatment, most notably the prevention of chemotherapy side effects and weight gain. All counseling sessions are conducted by RDs who are Certified Specialists in Oncology Nutrition (CSO) by the Academy of Nutrition and Dietetics [20], with additional training in exercise science. The intervention represents a core curriculum, using motivational counseling to address behavior strategies such as self-monitoring, goal setting, stimulus control, problem solving, and relapse prevention training. The intervention sessions are based on constructs of social-cognitive theory. The nutrition counseling promotes a predominantly plant-based diet with modifications to food texture, flavor and selection of tolerated nutrient-dense foods made on an individual basis to ensure adequate macronutrient and micronutrient food intake, and optimal glucose management.

Women receive our 16-chapter workbook adapted from the LEAN Study with skill building self-care topics across the trajectory of active treatment including: anticipating, tracking and managing treatment side effects; importance of hydration and managing liquid sugars; managing fatigue through exercise, meal timing and meal composition; managing expectations of others; comfort eating; the importance of nutrition and exercise with endocrine therapy and the late effects of treatment including cardiac health, lymphedema, fatigue, fear of food, and fear of recurrence. All 16 chapters are accompanied by a homework assignment; of these, recording daily food intake and physical activity are the most important.

We developed a Nutrition Impact Symptom (NIS) Management Recipe Book which contains 45 recipes kitchen-tested and adapted to the unique nutrition concerns of breast cancer patients to promote weight management and prevention of weight gain. Recipes are nutrient dense, low in added sugars, with soft textures to manage specific NIS of active treatment: xerostomia, mucositis, dysgeusia, dysphagia, nausea, vomiting, diarrhea, constipation, anorexia and fatigue.

### 3.3. Physical activity

The physical activity program, similar to our previous studies, relies on home-based exercise, with an emphasis on brisk walking and reaching a goal of 150 min per week of moderate-intensity exercise and 10,000 steps walked each day. Reducing sedentary behaviors is also encouraged through activities of daily living (see Table 2). The LEAN program increased physical activity levels by 116 min per week compared to 18 min per week among women randomized to usual care (*p* < 0.01) [18]. For the LEANer trial, we have enhanced the physical activity program offered in LEAN, by including a home-based progressive strength training program. Each participant randomized to intervention is given a set of dumbbells (3, 5 and 8 lbs.) to use at home. Patients are instructed during each counseling session to progressively increase repetitions and weight, individualized to their ability. The counseling is supported by a video of the LEANer Stretching, Core Exercises and Strength Training routine, which is posted on the study website.

All exercises are demonstrated and narrated by an American College of Sports Medicine Certified Cancer Exercise Trainer. Participants record the type and duration of exercise they do in their log book provided to them. We also include a tool-box approach, as was done in the Diabetes Prevention Program, where we provide participants with an optional 1-year membership to a YMCA that also offers the Livestrong Program [21] and Fitbits to motivate participants to increase their daily exercise.

### 3.4. Intervention goals

The goal of the intervention is for participants to adopt and practice the recommended dietary and physical activity guidelines. We measure adherence to the dietary guidelines by whether participants meet the Healthy Eating Index 2015 (HEI-2015), as it aligns with the USDA’s 2016 Dietary Guidelines for Americans (DGA), and has been found to be a valid and reliable measure of diet quality. We will also evaluate changes in intake of food group, total energy and energy from fat. Adherence to the physical activity guidelines is determined from a physical activity questionnaire completed at the follow-up visits. A combined “healthy behavior” score will be calculated for each individual and compared from baseline to end of study to assess change in adherence to nutrition and physical activity guidelines.

## 4. Usual care

During the trial, participants will only be contacted to complete the assessments. The usual care group has access to a RD at any time throughout treatment; referral is at the discretion of the treating oncologist. At the end of the study, usual care participants are offered an individualized diet and physical activity counseling session with a RD and receive a copy of the study materials. In addition, all participants, including the usual care participants, are referred to the Yale Cancer Center Survivorship Clinic or are able to access the Zakim Center for Integrative Therapies at Dana-Farber Cancer Institute.

## 5. Study assessments

### 5.1. Analysis of chemotherapy completion rate

At the completion of each woman’s chemotherapy session, data including date and dose of each drug administered, and reason for dose-adjustments and dose-delays are abstracted from the electronic medical record, or, if necessary, obtained from the treating oncologist. Chemotherapy completion rate will be assessed as the average relative dose-intensity (RDI) for the originally planned regimen based on standard formulas below [22]. Relative dose intensity (RDI) was defined as the ratio of actual dose intensity to projected dose intensity, expressing as a percentage or proportion of the amount of drug planned to deliver per weeks (mg/m2•week). The projected DI is calculated by:

$$\frac{Planned\;total\;dose\;(mg)}{BSA\times planned\;number\;of\;weeks\;on\;treatment}$$

where BSA stands for body surface area (m2). And the actual DI is calculated by:

$$\frac{total\;dose\;delivered\;(mg)}{BSA\times actual\;number\;of\;weeks\;on\;treatment}.$$

RDI will be calculated for each drug separately. The average RDI will be used as outcome measure for each patient.

We will measure the number and percent of patients who have dose-adjustments or delays, the reason for the dose-adjustment/dose delays (e.g. neuropathy or vacation) and the percent dose reduction for each participant.

### 5.2. Patient reported outcomes

Women are asked to complete self-administered study questionnaires prior to randomization, after completion of chemotherapy, 1- and 2-years post-randomization. Women are provided a link via email to the online questionnaires or given a hard copy version if preferred. Dietary intake over the past 3 months is assessed via a self-administered food frequency questionnaire [23]. Participants complete an interview-administered physical activity questionnaire. The past 3 months of physical activity, including the type, frequency and duration of 20 activities are assessed [24]. Additional patient reported assessments include endocrine therapy adherence, quality of life, neuropathy, patient reported outcomes, adherence to medication, lymphedema and employment experience. A subset of patients is asked to complete the 24-h dietary recall interviews.

### 5.3. Assessment of body composition, biomarkers and endocrine therapy adherence

At each of the four timepoints, women treated at Smilow Cancer Hospital Network are invited to complete in-person assessments, which are conducted at the Hospital Research Unit of Yale-New Haven Hospital. In-person procedures include: fasting blood draw to assess serum metabolic and inflammatory biomarkers, dual-energy X-ray absorptiometry scan to assess body composition, algometry to assess grip strength, skin carotenoid assessment [25], anthropometry (height, weight, waist and hip circumference) and urine collection for women who are prescribed aromatase inhibitors to assess compliance. Women are also asked to collect a stool sample. All assessments are described in Fig. 2 [31-37]

## 6. Statistical considerations

### 6.1. Power and sample size considerations

The sample size estimate was performed using PASS 12 (©2013 NCSS, LLC, Kayesvile, UT). The original calculation was based on chemotherapy completion rates reported in the Compliance in Adjuvant treatment in primary breast cancer Study (COMPAS) study [26]. By assuming similar completion rate in our control arm, we estimated a sample size of 250 subjects would be needed. Sample size reevaluation was conducted using the aggregated data of the first 51 LEANer participants who completed chemotherapy. This allowed us to have better accuracy in RDI variation in our patients. RDI was analyzed as continuous variable with a pooled standard deviation of 0.10. We re-estimated that a sample size of 86 subjects per arm (*n* = 172) will achieve 90% power to detect a 0.05 (or 5%) difference in RDI between two arms at significance level of 0.05, using two-sided two-sample equal-variance *t*-test.

For endocrine therapy adherence, the COMPAS study reported 48% of patients in the control group were adhering to AI therapy at 12 months [26]. We assume approximately 50% of patients randomized to usual care in our study will be adhering to endocrine therapy at two years post randomization (approximately one-year post-initiation of use). We estimate 70% of participants (i.e., 70% of 172 = 120) will be prescribed endocrine therapy; thus an *N* = 120 will also allow for 80% power to detect a 24% absolute increase in adherence in intervention group at alpha level of 0.05, or a 29% absolute increase at alpha level of **0.01.**

### 6.2. Statistical analyses plan

The study design is a two-arm superiority randomized controlled trial. The primary endpoint is RDI. RDI will be calculated using the methods described by Longo et al. [22]. The primary endpoint of RDI will be analyzed both as a continuous scale using a t-test, and as a dichotomized outcome using 85% or 95% cut-off for Mantel-Haensel chi-square test while controlling for stratification factors. Group differences in chemotherapy completion will be analyzed with binary logistic regression analysis; whether or not having dose reduction during the period of chemotherapy treatment (the period between T0 and T1) will be the dichotomized dependent variable.

The secondary outcomes including endocrine therapy adherence, body compositions and biomarkers. The outcome of endocrine therapy adherence will be assessed at the 2-year visit (as most, if not all, participants prescribed endocrine therapy will have been taking it for at least one year at this 2-year visit, allowing us to assess adherence to endocrine therapy). An undetectable urine AI level will classify them as nonadherent to AIs [26]. Our secondary measure of endocrine therapy adherence will be based on the following questions: (1) “In the past month, how often did you take your medications as the doctor prescribed?” Possible responses are: all of the time (100%), nearly all of the time (90%), most of the time (75%), about half the time (50%), or less than half the time (<50%). Nonadherence is defined as 75% of the time or less. (2) “In the past month, how often did you forget to take 1 or more of your prescribed medications?” Possible responses were never, once in the past month, 2 to 3 times in the past month, once per week, several times per week, and nearly every day. Nonadherence is defined as forgetting to take prescribed medications once per week or more. (3) “In the past month, how often did you decide to skip 1 or more of your prescribed medications?” Possible responses were the same as for question 2. Nonadherence is defined as deciding to skip medications once per week or more [27]. Participants who report that they have discontinued taking tamoxifen or AI will also be classified as nonadherent to endocrine therapy. For the endocrine therapy aim, we will compare the crude adherence rates using simple logistic regression. Multivariate logistic regression will serve as supportive analyses including covariate adjustments – which are similar to the primary analysis above.

The other secondary outcomes will be performed using Mixed Model Repeated Measures analysis incorporating Analysis of Covariance, where each woman’s changes in outcome (follow-up measure – baseline) will be modeled as a function of treatment and time (fixed effects) with the baseline value and stratification factors as covariates. Prior to fitting models, we will perform exploratory data analyses focusing on the distributions of biological markers by time and intervention group, assessing the appropriateness of log-transformation.

All hypotheses will be tested according to the intention-to-treat philosophy in which all randomized participants will be grouped according to their intervention assignment at randomization, regardless of compliance or adherence to the study. Statistical significance will be defined as *p* < 0.05, 2-sided. Since we have multiple outcomes, we will consider using a Bonferroni correction.

Age, menopausal status, race/ethnicity, BMI at diagnosis, disease stage, type of surgery, type of chemotherapy, neoadjuvant/adjuvant chemotherapy, radiation therapy, HR status, HER2 status, endocrine therapy, reconstructive surgery, type 2 diabetes, smoking status and study site will be examined as covariates. We will explore effect modification by menopausal status at diagnosis, hormone receptor status, and endocrine therapy. We will further explore intervention effects on study endpoints by stratifying by adherence to diet and physical activity guidelines with a multi-level category (e.g., met diet recommendations, met physical activity, met both diet and physical activity recommendations, or met neither recommendation). This analysis will allow us to better understand the role of diet vs. exercise on study endpoints.

Although we do not anticipate an appreciable number of subjects lost-to-follow-up because of our plan of following patients during their medical oncology visits, the impact of missing data will be evaluated in the analysis. Bivariate logistic regression will be used to identify baseline variables associated with missingness. The primary analysis assumes missing data are missing at random (MAR). Under the MAR missing data mechanism, the probability of loss-to-follow-up depends only on the observed data. Mixed effects model analysis using maximum-likelihood estimation method is an efficient way to handle MAR data [38]. Non-random or informative loss-to-follow-up occurs when the missing data are dependent on the unobserved missing outcome values, latent and/or instrumental variables. Sensitivity analyses using methods for not missing at random (NMAR) data will be considered.

## 7. Data management

Participants will complete online questionnaires using a secure HIPAA-compliant service (Qualtrics) or via mailed paper questionnaires. Study data is stored on a secure server which is maintained by Yale University Medical School. Database access is limited by individual ID and password. The primary outcome data is entered in duplicate by different staff members and is reconciled by a third staff member.

## 8. Data and safety monitoring

As the study is considered minimal risk, the principal investigator (TS) is responsible for monitoring the data, assuring protocol compliance, and conducting the safety reviews. During the review process the principal investigator will evaluate whether the study should continue unchanged, require modification/amendment, or close to enrollment. A log of adverse events will be maintained by the study staff and reviewed on a regular basis by the principal investigator (TS), serious adverse events will be reported as per IRB guidance. Adverse events will be categorized and summarized by intervention arms using counts and proportions. The exact binomial test will be considered. The study protocol will be reviewed on an annual basis by the relevant IRBs. Protocol changes will be communicated to relevant parties via weekly team meetings and timely email communications. A routine review will be conducted annually by the Office of Quality Assurance & Monitoring, Yale Center for Clinical Investigation.

## 9. Discussion

Higher levels of physical activity and lower body mass index (BMI) have been shown to be associated with better adherence to chemotherapy completion rate and increased pathological response rate, respectively, in women with breast cancer receiving neoadjuvant chemotherapy [15]. Thus, lifestyle behavioral interventions to improve diet and increase physical activity have the potential to improve response to chemotherapy, potentially via improvement in compliance to chemotherapy.

Van Waart and colleagues evaluated the effect of a home-based, low-intensity physical activity program (Onco-Move) and a supervised, moderate- to high-intensity, combined resistance and aerobic exercise program (OnTrack) compared to usual care on chemotherapy completion rates [28]. Based on their medical record review, the planned chemotherapy regimens and schedules of the three groups were similar and included combinations of anthracyclines, taxanes, alkylating agents, and antimetabolites. In total, 61 of 230 patients (27%) required chemotherapy dose adjustments, with a smaller percentage of OnTrack participants requiring chemotherapy dose adjustments (12%) than those in the Onco-Move (34%) or usual care group (34%), *p* = 0.002. The average dose reduction among those who required chemotherapy adjustment in OnTrack and Onco-Move was 10%, compared with 25% in usual care; *p* = 0.014, with neuropathy being the primary reason for dose adjustments. Similarly, Courneya and colleagues’ conducted an exercise trial in 242 breast cancer survivors initiating adjuvant chemotherapy [29]. Women were randomly assigned to usual care (*n* = 82), supervised resistance exercise (n = 82), or supervised aerobic exercise (*n* = 78) for the duration of their chemotherapy (median, 17 weeks; 95% CI, 9 to 24 weeks). While the primary endpoint was quality of life, a secondary endpoint was chemotherapy completion rate. Adjusted mixed-models analyses indicated that resistance exercise was superior to usual care for improving chemotherapy completion rate (*p* = 0.033). Relative dose-intensity was 84% in the usual care group compared with 90% in the resistance exercise group (p = 0.033) and 87% in the aerobic exercise group (*p* = 0.266). The percentage of participants who received at least 85% of their planned dose-intensity was 66% in the usual care group compared with 78% in the resistance exercise group (*p* = 0.082) and 74% in the aerobic exercise group (*p* = 0.241).

Carayol et al. [30] recently reported a beneficial effect of a physical activity and nutrition intervention vs. usual care on patient reported outcomes in 143 early stage breast cancer patients receiving chemotherapy. The intervention included thrice-weekly moderate-intensity mixed aerobic and resistance exercise sessions and 9 nutrition consultations. Chemotherapy completion rate was reported as a secondary endpoint. No difference between groups was seen for the relative dose intensity (96.7% in the intervention arm versus 96% in usual care arm; *p* = 0.39); however, a higher percentage of patients in the intervention arm (78.8%) received more than 95% of their planned relative dose intensity compared to the usual care arm (65.1%), *p* = 0.08. While Carayol’s trial is suggestive of an effect of nutrition and exercise on improving chemotherapy completion rate, given the secondary aim, it is likely that reasons for chemotherapy dose delays or adjustments were not taken into consideration. To our knowledge, the LEANer study is the first randomized nutrition and exercise intervention to be administered during chemotherapy with the primary aim of improving chemotherapy completion rates. Our approach includes a detailed EMR review and real-time follow-up with the oncologist to document reasons for chemotherapy dose delays or adjustments.

In addition, improving nutrition and physical activity levels show promise for improving adherence to endocrine therapy, in turn improving breast cancer survival. We completed a randomized controlled exercise trial on side effects of aromatase inhibitors including arthralgias and found exercise to improve arthralgias by 29% compared to a 3% worsening in usual care (*p* < 0.001). Arthralgias are a common and primary reason for poor adherence to aromatase inhibitors. The LEANer study will examine the impact of nutrition and exercise on side effects of endocrine therapy as well as adherence to endocrine therapy.

Ultimately, if our study finds that adopting and following the dietary and physical activity guidelines improves adherence to chemotherapy and endocrine therapy, serum biomarkers, body composition and patient reported outcomes, then lifestyle behavioral counseling may become a standard of care in breast cancer treatment, in turn impacting obesity rates, treatment-related side effects and improved disease-free survival.

## Figures and Tables

**Table 1:** Eligibility criteria.

Inclusion criteria
• Diagnosed with Stage I-III breast cancer
• Adjuvant or neoadjuvant chemotherapy is part of breast cancer treatment
• Physically able to walk
• Able to complete forms and understand instructions in English
• Agrees to be randomly assigned to either intervention or usual care group
Exclusion criteria
• Received their second chemotherapy infusion
• Eating 7 or more fruits and vegetables a day
• Doing 150 or more minutes per /week of moderate to vigorous intensity physical activity or 75 min/week of vigorous-intensity physical activity
• Are pregnant or intending to become pregnant in the next year
• Recent (past year) stroke/myocardial infarction or congestive heart failure
• Presence of dementia or major psychiatric disease
• Are malnourished (PG-SGA assessment)
• Participating in a meal replacement weight loss program

**Table 2:** Lifestyle guidelines.

Dietary guidelines
• Eat a combination of 7+ fruits and/or vegetables servings/day
• Reduce simple sugars
• Limit consumption of processed and red meats to ≤18 oz/week
• Limit alcohol consumption to 1drink/day or 8 drinks/week
Physical activity guidelines
• 150+ min/week of moderate to vigorous intensity physical activity or 75 min/week of vigorous-intensity physical activity
• Twice-weekly strength training
• Reduce sedentary time

## Data Availability

T.S. and M.L.I. are the guarantors of the research data and material described in this manuscript. Data sharing is not applicable to this article as no datasets were generated or analyzed during the current study.

## Abbreviations:

ACS: American Cancer Society
AI: Aromatase Inhibitor
AICR: American Institute of Cancer Prevention
BCPT: Breast cancer prevention trial
BMI: Body Mass Index
COMPAS: Compliance in adjuvant treatment in primary breast cancer study
CPS-II: Cancer prevention study-II
CSO: Certified specialist in oncology nutrition
DGS: Dietary guidelines for Americans
DHHS: U.S. Department of Health and Human Services
DXA: Dual energy X-ray absorptiometry
EORTC QLQ-CIPN: European Organization for Research and Treatment of Cancer, quality of life questionnaire-chemotherapy-induced peripheral neuropathy
IRB: Institutional Review Board
HEI-2015: Healthy eating index 2015
HR: Hormone receptor
LEANer: Lifestyle Exercise and Nutrition Early after Diagnosis
RDI: Relative dose-intensity
NCI: National Cancer Institute
RD: Registered dietitian
LEAN: Lifestyle, Exercise and Nutrition
MAR: Missing at random
NIS: Nutrition impact symptom
NMAR: Not missing at random
PROMIS: Patient-reported outcomes measurement information system
VITAL: Vitamins and lifestyle study
WCRF: The World Cancer Research Fund
